# Associations of sleep duration, daytime napping, and snoring with depression in rural China: a cross-sectional study

**DOI:** 10.1186/s12889-023-16479-w

**Published:** 2023-08-11

**Authors:** Xueyao Zhang, Guangxiao Li, Chuning Shi, Yingxian Sun

**Affiliations:** 1 Department of Cardiology, First Hospital of China Medical University, NO.155 Nanjing North Street, Heping District, Shenyang, 110001 China; 2 Department of Medical Record Management, First Hospital of China Medical University, NO.155 Nanjing North Street, Heping District, Shenyang, 110001 China

**Keywords:** Depression, Sleep duration, Napping, Snoring, Rural China

## Abstract

**Background:**

Most adult patients with depression complain about sleep symptoms, including insufficient and excessive sleep. However, previous studies investigating the impact of sleep duration on depression have yielded conflicting results. Therefore, this study aimed to analyse the link between depression and sleep duration, daytime napping, and snoring among rural Chinese adults.

**Methods:**

A cross-sectional study was conducted with 9104 individuals. Interviews were conducted with the participants regarding their sleep patterns and their daytime napping routines. The individuals were then assessed for depression using the Patient Health Questionnaire-9. The risk of depression was assessed using a multifactor binary logistic regression analysis. A generalized additive model was used to evaluate the nonlinear relationship between depression and sleep duration/nap time. Additionally, subgroup analysis was conducted to investigate the correlation between sleep duration, daytime napping, snoring, and depression.

**Results:**

Less than 6 h or more than 8 h of nighttime sleep, daytime napping for more than 1 h, and snoring were all significantly associated with an increased risk of depression. A U-shaped relationship was found between the duration of nighttime sleep and depression. In addition, we found that the nighttime duration of sleep, daytime naps, and snoring had a significant combined effect on the risk of depression. The subgroup analysis further revealed that lack of sleep at night significantly increased the risk of depression in all subgroups. However, snoring and excessive nighttime sleep and napping were only associated with the risk of depression in some subgroups.

**Conclusions:**

Lack of nighttime sleep (short sleep duration), excessive sleep, and napping for more than one hour during the day were associated with a high risk of depression and had a combined effect with snoring.

## Background

*Depression* is a common disabling mental disease that creates an economic burden on global public health. Depression is regarded as China’s second-leading cause of disability [1], and its prevalence is increasing rapidly with China’s economic growth and societal changes [2]. According to recent research, the prevalence of depression is negatively correlated with economic income and employment [3], and the treatment gap for common mental health diseases worldwide due to economic reasons exceeds 80% [4]. Furthermore, most patients with depression report social disorders and have a low rate of diagnosis and treatment, and the situation is challenging in rural areas in relatively undeveloped economies [1]. However, few studies have investigated modifiable factors that influence depression in rural or agricultural areas. Therefore, identifying risk factors and providing primary health care and interventions remains challenging and essential [5].

Adequate and high-quality sleep at an appropriate time helps maintain physical and mental health [6]. Epidemiological studies have observed that sleep duration is related to cardiovascular events [7, 8], emotional disorders, and mortality [9]. Up to 90% of adult patients with depression complain of sleep problems, including insufficient and excessive sleep [10]. In recent years, although the impact of sleep duration on the development of depression has been investigated, the conclusions of different studies have been conflicting. For instance, one study demonstrated a significant correlation between depression and short sleep duration [11]. Another found that short sleep duration (including difficulty in sleep maintenance and early awakening in the morning) was not related to depression [12].

Moreover, the link between prolonged sleep duration and depression is also controversial. Although, contrary to popular belief, previous results have not shown a link between depression and self-reported excessive sleepiness or objectively measured total sleep duration [13, 14], another study has suggested that people who have a high or low amount of sleep have an increased risk of depression [15]. The underlying cause of these conflicting results is unknown, but sampling different ethnic groups, and even urban and rural differences, may contribute to these discrepancies. To clarify these issues, the link between depression and sleep duration needs further research. In addition, most studies that have evaluated the relationship between sleep duration and depression have not considered snoring and habitual daytime napping, which affect sleep quality and duration [16]. Among them, snoring has been confirmed to be clearly associated with depression [17], while biochemical indicators (especially blood lipids, blood sugar, uric acid, etc.) also tend to be considered as possibly related to depression [18–21]. A better understanding of the impact of modifiable sleep conditions on depression could reduce the considerable economic and health burden of rural depression prevention and control. Therefore, we investigated how depression was related to sleep duration (including excessive sleep, insufficient sleep, and daytime napping) and snoring.

## Methods

**Fig. 1:**
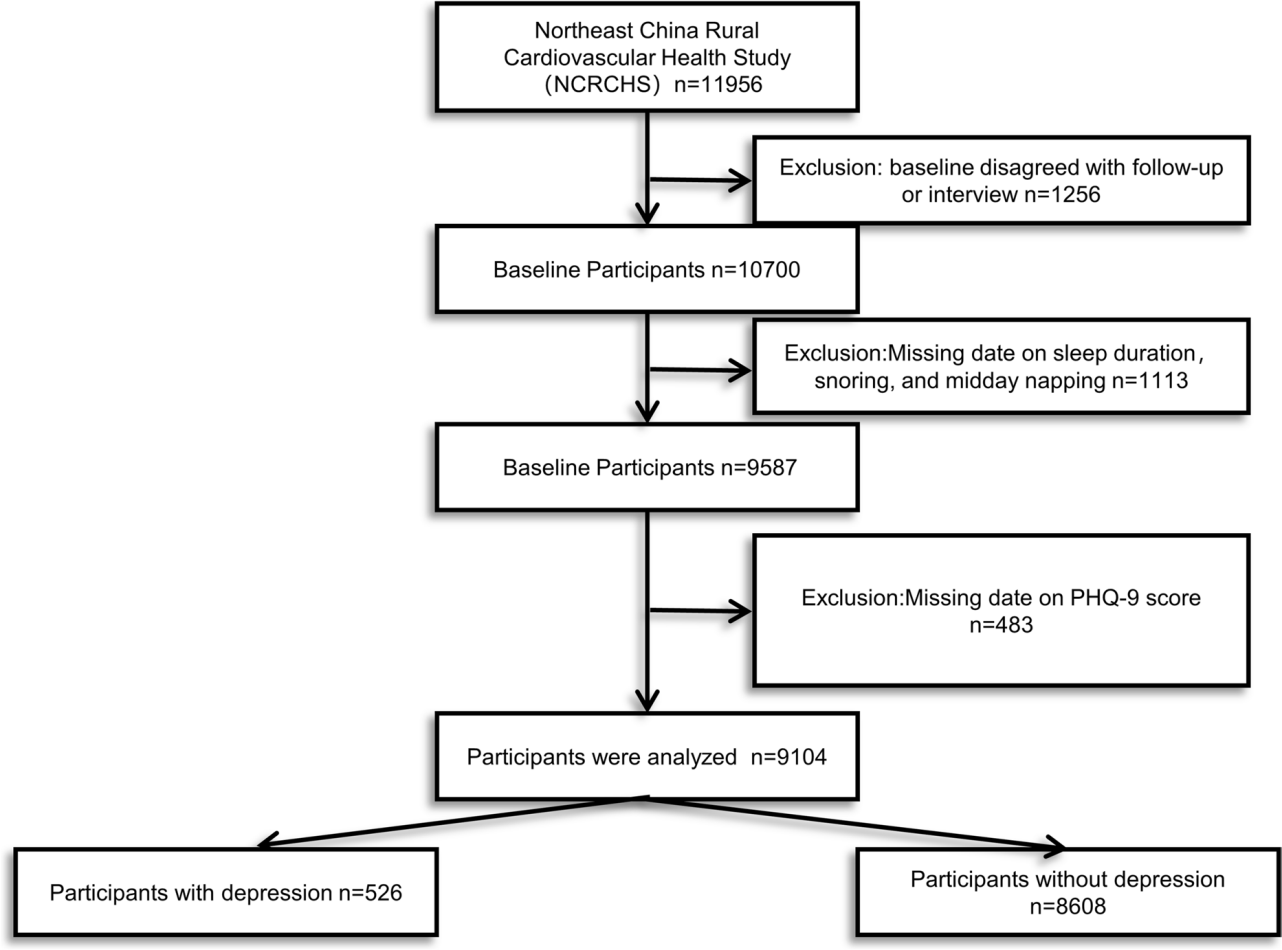
Participant screening flowchart. A total of 9104 participants were ultimately included in the analysis, after applying exclusion criteria

The study participants were from a previously reported population-based prospective cohort study (Northeast China Rural Cardiovascular Health Study) [22]. The data analysed were obtained from its cross-sectional survey. This study was conducted between February 2012 and January 2013, using multistage, stratified, and random clustering sampling. In the first stage, Dawa in the east, Zhangwu in the south, and Liaoyang in the north were selected from different locations in Liaoning Province in Northeast China, and 8–10 rural communities were randomly selected from each town mentioned above (26 rural villages in total). Of the 11,956 eligible resident villagers (who were required to be ≥ 35 years old) invited to participate, 10,700 participants (response rate: 89.5%) agreed and were eligible to participate in our follow-up study. Their baseline information was collected. The exclusion criteria included patients with malignant tumours, pregnancy, and severe mental disorders who could not cooperate with the investigation or those who planned to move in a short time. In the data analysis, 1596 participants with missing sleep information and depression assessment data were also excluded (Fig. 1, flowchart).

### Assessment of covariates

In this study, trained researchers from the Centres for Disease Control in Liaoning Province and the China Medical University interviewed each participant face-to-face using a standardized questionnaire. In addition, researchers simultaneously collected clinical, demographic (age and gender), and lifestyle information such as smoking, alcohol consumption, physical activity intensity, and medical history (cardiovascular-related diseases and chronic disease drug use) from all participants. Detailed definitions and collection methods have been reported elsewhere [23]. Body mass index (BMI) was calculated as weight (kg) divided by the square of height (m^2^). When blood pressure was measured (sphygmomanometer: Omron HEM-907), the participants were seated and were advised to rest for more than 10 min and avoid stimulating beverages before the measurement. The average of three measurements (with an interval of more than 10 s between each) was used as the systolic blood pressure (SBP) and diastolic blood pressure (DBP). Hypertension diagnostic criteria were mean SBP ≥ 140 mmHg, DBP ≥ 90 mmHg, self-reported hypertension, or currently receiving antihypertensive treatment. After 12 h of fasting, participants were tested for fasting plasma glucose (FPG), total cholesterol, triglycerides, low-density lipoprotein cholesterol (LDL-C), uric acid (UA), serum creatinine (Cr), high-density lipoprotein cholesterol (HDL-C), and other tests (Olympus AU 640, Tokyo, Japan), as described previously [24]. The diagnostic standard of diabetes was FPG ≥ 7.0 mmol/L or self-statement of previous diagnosis by a doctor or receiving hypoglycaemic treatment [25]. The estimated glomerular filtration rate (eGFR) was based on the CKD-EPI formula standard [26]. Uric acid (UA) level above 420 mg/dL was defined as hyperuricemia [27]. Dyslipidaemia was diagnosed according to the National Cholesterol Education Program Adult Treatment Panel III as total cholesterol ≥ 6.21 mmol/L, triglyceride ≥ 2.26 mmol/L, low-density lipoprotein ≥ 4.16 mmol/L (high), and high-density lipoprotein ≤ 1.03 mmol/L (low) [28]. Diabetes [18], hypertension [19], dyslipidaemia [20], hyperuricemia [21] and other risk factors are associated with depression, so these variables are selected as covariates in the form of continuous variables.

### Assessment of sleep duration and habits

Sleep duration (the quantity of time that a participant sleeps) and daytime napping habits were assessed by conducting face-to-face interviews. Daytime napping was assessed by asking participants: ‘Do you usually take a nap during the daytime?’ Answer options were *Yes* or *No*; if yes, the participant was asked to ‘please specify the duration’. The napping duration was divided into three groups: <0.5, 0.5–1, and > 1 h. Snoring was determined based on the response to the question, ‘Do you snore while sleeping?’ The response options were *No* or *Yes*.

### Assessment of depression

The Patient Health Questionnaire-9 (PHQ-9), the most common self-administered depression assessment, is widely used to screen for depression in primary health centres [29]. Each of the nine DSM-IV criteria [30] is scored from ‘0’ (*not at all*) to ‘3’ (*almost every day*). The study participants were asked how often they had been troubled by depressive symptoms in the past two weeks. The depression scores on the PHQ-9 scale range from 0 to 27. They are categorized into three groups: *mild* (≥ 5), *moderate* (≥ 10), or *severe* (≥ 15). The PHQ-9 is a reliable and effective indicator to measure the severity of depression [31], with a depression threshold set at 10 [32].

### Statistical analyses

Descriptive statistics of categorical variables were expressed as counts (%); continuous variables were reported as mean ± standard deviation (SD) or median (interquartile range [IQR]). As applicable, differences between groups were assessed using a *t*-test, one-way ANOVA, and non-parametric or chi-square tests. Multivariate binary logistic regression analysis was used to calculate the odds ratio (OR) and 95% confidence interval (CI) to assess the risk of depression. The following models were developed: Model 1 was raw; Model 2 added adjustments based on race, age, and sex; Model 3 added physical activity intensity, smoking, drinking, marriage, education level, and annual per capita income to Model 2; and Model 4 adjusted for race, age, sex, physical activity intensity, smoking, drinking, marriage, education level, annual per capita income, SBP, DBP, FPG, UA, triglycerides, HDL-C, LDL-C, total cholesterol, eGFR, BMI, and multiple sleep variables (snoring, sleep duration, and daytime napping). The nonlinear relationship between depression and sleep duration/nap duration was evaluated through a generalized additive model (GAM). The *gam* function in the R software *mgcv* package was used for smoothed curve fitting [33]. In addition, stratified analyses were then carried out on the correlations between sleep duration, daytime napping, snoring, and depression; the reference group was medium sleep duration (7–7.9 h) and short nap duration (< 0.5 h). SPSS 25.0 and R software (3.6.3) were used to complete the statistical analysis. *P* values < 0.05 were considered statistically significant.

## Results

### Demographic characteristics

A total of 9104 participants (including 4127 males) with an average age of 53.51 ± 10.37 years were included in the analysis. The baseline characteristics of the study participants are listed in Table 1. The prevalence of depression was 5.78% (526/9104). Among the 9104 participants, 54.5%, 36.5%, and 9.0% reported a sleep duration of < 6, 6–8, and > 8 h, respectively, and 33.7% (3068/9104) reported a daytime napping habit. Differences in participants’ age, sex, blood pressure, diabetes, blood lipid level, uric acid, depression score and prevalence rate, marriage, education, income level, exercise, and physical activity intensity were found among the sleep duration groups. However, no significant difference in snoring was detected. In addition, compared with the non-napping group, the daytime napping group had a higher proportion of older men belonging to Han nationality. They had greater diabetes prevalence and higher blood lipids, serum uric acid, and depression scores, and many were unmarried. They reported lower levels of physical activity intensity, a lower proportion of exercise, and a higher proportion of snoring. However, no statistically significant difference was found between the two groups in hypertension, sleep duration, or depression.

**Table 1:** Baseline characteristics of participants

Variables	Sleep duration			*P*-value	Daytime napping		*P*-value
	< 6 h(4959)	6–8 h (*n* = 3326)	> 8 h (*n* = 819)		No(*n* = 6036)	Yes(*n* = 3068)	
Age (years)	52.03 ± 10.25	55.92 ± 10.03	52.71 ± 10.64	< 0.001	52.65 ± 10.04	55.22 ± 10.78	< 0.001
Male, n(%)	2336 (47.11%)	1395 (41.94%)	396 (48.35%)	< 0.001	2495 (41.34%)	1632 (53.19%)	< 0.001
Han nationality, n(%)	4656 (93.89%)	3171 (95.34%)	767 (93.65%)	0.012	5668 (93.90%)	2926 (95.37%)	0.004
BMI (kg/m^2^)	24.92 ± 3.68	24.59 ± 3.67	25.08 ± 3.54	< 0.001	24.87 ± 3.64	24.70 ± 3.71	0.035
Hypertension, n(%)	2412 (48.64%)	1761 (52.95%)	471 (57.51%)	< 0.001	3039 (50.35%)	1605 (52.31%)	0.076
Diabetes, n(%)	456 (9.20%)	369 (11.09%)	75 (9.16%)	0.014	560 (9.28%)	340 (11.08%)	0.006
TC(mmol/L)	5.18 ± 1.06	5.32 ± 1.11	5.29 ± 1.13	< 0.001	5.26 ± 1.11	5.20 ± 1.05	0.007
TG(mmol/L)	1.19 (0.84–1.81)	1.27 (0.90–1.91)	1.23 (0.91–1.90)	0.025	1.19 (0.85–1.79)	1.31 (0.91–2.01)	< 0.001
LDL-C(mmol/L)	2.92 ± 0.82	2.96 ± 0.83	3.03 ± 0.92	< 0.001	2.98 ± 0.86	2.87 ± 0.78	< 0.001
HDL-C(mmol/L)	1.43 ± 0.39	1.41 ± 0.38	1.45 ± 0.42	0.006	1.44 ± 0.39	1.38 ± 0.38	< 0.001
UA(mg/dL)	4.84 ± 1.40	4.85 ± 1.40	4.67 ± 1.27	0.003	4.71 ± 1.35	5.07 ± 1.43	< 0.001
Marriage, n(%)				< 0.001			0.002
With spouse	4594 (92.64%)	2941 (88.42%)	756 (92.31%)		5536 (91.72%)	2755 (89.80%)	
No spouse	365 (7.36%)	385 (11.58%)	63 (7.69%)		500 (8.28%)	313 (10.20%)	
Education, n(%)				< 0.001			< 0.001
Illiteracy	354 (7.14%)	383 (11.51%)	81 (9.89%)		598 (9.91%)	220 (7.17%)	
Middle school or below	4101 (82.70%)	2694 (81.00%)	679 (82.91%)		4886 (80.95%)	2588 (84.35%)	
High school or above	504 (10.16%)	249 (7.49%)	59 (7.20%)		552 (9.14%)	260 (8.48%)	
Annual income(CNY), n(%)				< 0.001			< 0.001
≤ 5000	495 (9.98%)	481 (14.46%)	99 (12.09%)		611 (10.12%)	464 (15.12%)	
5000– 20,000	2710 (54.65%)	1889 (56.80%)	442 (53.97%)		3403 (56.38%)	1638 (53.39%)	
≥ 20,000	1754 (35.37%)	956 (28.74%)	278 (33.94%)		2022 (33.50%)	966 (31.49%)	
Physical labor intensity, n(%)				< 0.001			< 0.001
Light	1553 (31.32%)	1364 (41.01%)	282 (34.43%)		2201 (36.46%)	998 (32.53%)	
Moderate	917 (18.49%)	615 (18.49%)	149 (18.19%)		1123 (18.61%)	558 (18.19%)	
Severe	2489 (50.19%)	1347 (40.50%)	388 (47.38%)		2712 (44.93%)	1512 (49.28%)	
Exercise, n(%)				0.01			< 0.001
No	3986 (80.38%)	2584 (77.69%)	657 (80.22%)		4912 (81.38%)	2315 (75.46%)	
Yes	973 (19.62%)	742 (22.31%)	162 (19.78%)		1124 (18.62%)	753 (24.54%)	
Snoring, n(%)				0.645			< 0.001
No	3058 (61.67%)	2031 (61.06%)	492 (60.07%)		3843 (63.67%)	1738 (56.65%)	
Yes	1901 (38.33%)	1295 (38.94%)	327 (39.93%)		2193 (36.33%)	1330 (43.35%)	
Night sleep(hours)	7.58 ± 0.51	5.26 ± 0.98	9.46 ± 0.73	< 0.001	6.89 ± 1.54	6.93 ± 1.53	0.193
PHQ-9 score	2.24 ± 2.97	4.14 ± 4.20	2.57 ± 3.48	< 0.001	2.89 ± 3.62	3.11 ± 3.64	0.008
Depression, n(%)				< 0.001			0.135
No	4813 (97.06%)	2991 (89.93%)	774 (94.51%)		5703 (94.48%)	2875 (93.71%)	
Yes	146 (2.94%)	335 (10.07%)	45 (5.49%)		333 (5.52%)	193 (6.29%)	

*Abbreviations:BMI* Body mass index, *TC* Total cholesterol, *TG* Triglycerides, *LDL-Cl* Low-density lipoprotein cholesterol, *HDL-C* High-density lipoprotein cholesterol, *UA* Uric acid, *PHQ-9* Patient Health Questionnaire-9

### Association between sleep duration, daytime napping, snoring, and depression

Table 2 shows the relationship between depression and sleep duration, daytime napping, and snoring. Compared with a sleep duration of 7–8 h, in the fully adjusted model (Model 4), participants with a nighttime sleep duration of fewer than 5 h had the highest risk of depression (OR 4.66; 95% CI 3.72–5.84); the groups with a sleep duration of 5–5.9 h and > 8 h also had a higher risk of depression (OR 1.82 and 2.44, respectively), while the groups with a nighttime sleep duration of 6–6.9 h had no significant risk of depression (*P* = 0.086). Compared with the group that did not nap during the daytime, the risk of depression did not increase in those with napping habits (OR 1.19; 95% CI 0.98–1.44; *P* = 0.081). However, we further discovered that, compared with the group with daytime napping habits of < 0.5 h, napping for more than 1 h was associated with an increased risk of depression (OR 1.63; 95% CI 1.20–2.23; *P* = 0.002). In addition, each adjusted model showed that snoring was associated with an increased risk of depression (*P* < 0.001).

**Table 2:** Association between sleep status and depression in multiple regression model

	Statistics	Model 1	*P*-value	Model 2	*P*-value	Model 3	*P*-value	Model 4	*P*-value
		OR (95% CI)		OR (95% CI)		OR (95% CI)		OR (95% CI)	
**Sleep duration (hours)**									
7–8	1790 (19.66%)	Ref		Ref		Ref		Ref	
<5	1039 (11.41%)	5.87 (4.73, 7.28)	< 0.0001	5.04 (4.04, 6.29)	< 0.0001	4.68 (3.74, 5.86)	< 0.0001	4.66 (3.72, 5.84)	< 0.0001
5-5.9	3920 (43.06)	2.01 (1.55, 2.61)	< 0.0001	1.88 (1.45, 2.44)	< 0.0001	1.84 (1.41, 2.39)	< 0.0001	1.82 (1.40, 2.37)	< 0.0001
6-6.9	1536 (16.87%)	1.54 (0.96, 2.46)	0.0709	1.53 (0.96, 2.45)	0.0761	1.49 (0.93, 2.39)	0.0982	1.51 (0.94, 2.43)	0.0858
>8	819 (9.00%)	2.44 (1.56, 3.81)	< 0.0001	2.47 (1.58, 3.87)	< 0.0001	2.41 (1.53, 3.78)	0.0001	2.44 (1.55, 3.84)	0.0001
**Daytime napping**									
No	6036 (66.30%)	Ref		Ref		Ref		Ref	
Yes	3068 (33.70%)	1.15 (0.96, 1.38)	0.135	1.20 (1.00, 1.45)	0.0542	1.19 (0.98, 1.44)	0.077	1.19 (0.98, 1.44)	0.0805
**Daytime napping(hours)**									
< 0.5	6766 (74.32%)	Ref		Ref		Ref		Ref	
0.5-1	1707 (18.75%)	0.75 (0.58, 0.96)	0.0233	0.81 (0.63, 1.05)	0.1062	0.81 (0.63, 1.05)	0.1121	0.82 (0.63, 1.06)	0.1265
> 1	631 (6.93%)	1.47 (1.09, 1.98)	0.0114	1.71 (1.26, 2.32)	0.0006	1.65 (1.21, 2.25)	0.0015	1.63 (1.20, 2.23)	0.002
**Snoring**									
No	5581 (61.30%)	Ref		Ref		Ref		Ref	
Yes	3523 (38.70%)	1.43 (1.20, 1.71)	< 0.0001	1.46 (1.22, 1.75)	< 0.0001	1.48 (1.23, 1.77)	< 0.0001	1.56 (1.30, 1.88)	< 0.0001

Model 1: raw model; Model 2 adjusted for race, age, and sex; Model 3 adjusted for race, age, sex, physical activity intensity, smoking, drinking, marriage, education level, and annual per capita income; Model 4 adjusted for race, age, sex, physical activity intensity, smoking, drinking, marriage, education level, annual per capita income, SBP, DBP, FPG, UA, triglycerides, HDL-C, LDL-C, total cholesterol, eGFR, BMI, snoring, sleep duration, and daytime napping

### Smoothed curve fitting and combined effect with snoring

**Fig. 2:**
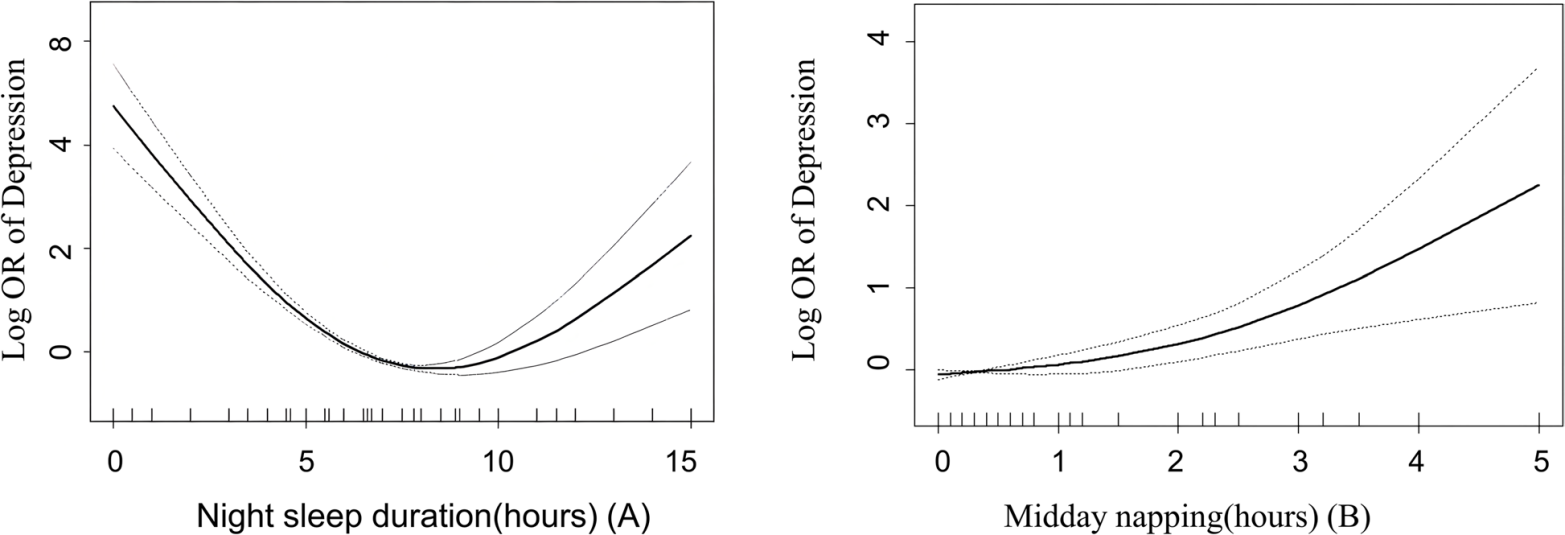
Smoothed curve fit of sleep duration, daytime napping, and depression after adjustment for potential confounding factors: race, age, sex, physical activity intensity, smoking, drinking, marriage, education level, annual per capita income, SBP, DBP, FPG, UA, triglycerides, HDL-C, LDL-C, total cholesterol, eGFR, BMI, snoring, sleep duration, and daytime napping. There was a U-shaped relationship between sleep duration and depression, and the risk of depression increased with the duration of daytime napping

**Fig. 3:**
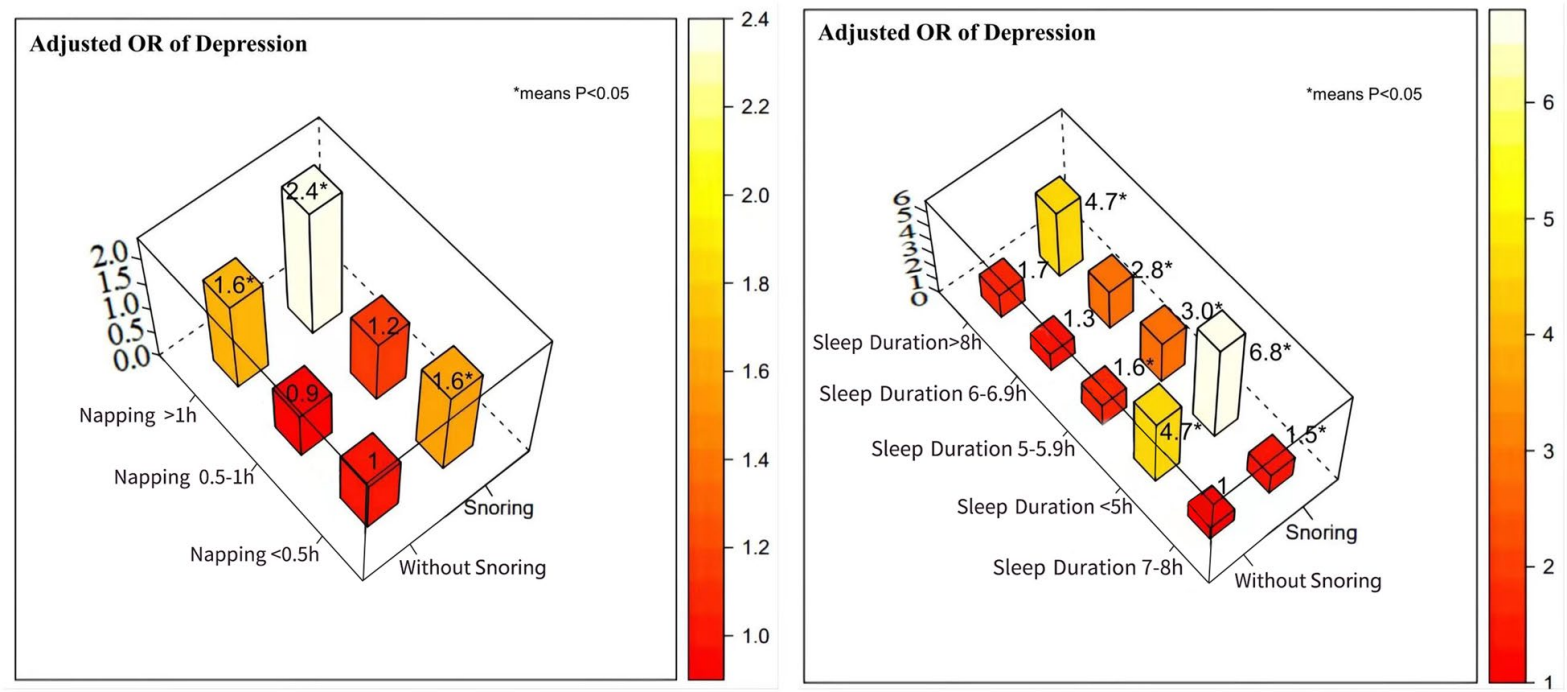
The effects of sleep duration or napping combined with snoring on the risk of depression. For nap duration, nap > 1 h combined with snoring increased the risk of depression (nap < 0.5 h without snoring as the reference group). Compared with those who reported moderate sleep duration (7–8 h) and did not snore, participants with short sleep duration (< 5 h) combined with snoring had a significantly increased risk of depression, while snoring increased the risk of depression, regardless of sleep duration. Each subgroup analysis was adjusted, if not stratified (*means, *P* < 0.05)

Figure 2 shows the smoothed fit curve of sleep duration, daytime napping, and depression after adjusting for potential confounding factors (the adjustment variables were the same as those in Model 4). There was a U-shaped relationship between sleep duration and depression, and the risk of depression increased with the duration of daytime napping. In addition, we also explored the combined effects of nap and nighttime sleep durations combined with snoring on depression risk (Fig. 3). For nap duration, using a nap < 0.5 h without snoring as the reference group, a nap > 1 h combined with snoring increased the risk of depression (OR 2.4; 95% CI 1.5–3.7; *P* < 0.001). Compared with those who reported moderate sleep duration (7–8 h) and did not snore, participants with short sleep duration (< 5 h) combined with snoring had a significantly increased risk of depression (OR 6.8; 95% CI 4.9–9.3; *P* < 0.001), while snoring increased the risk of depression, regardless of nighttime sleep duration (*P* < 0.05).

### Subgroup analysis

**Fig. 4:**
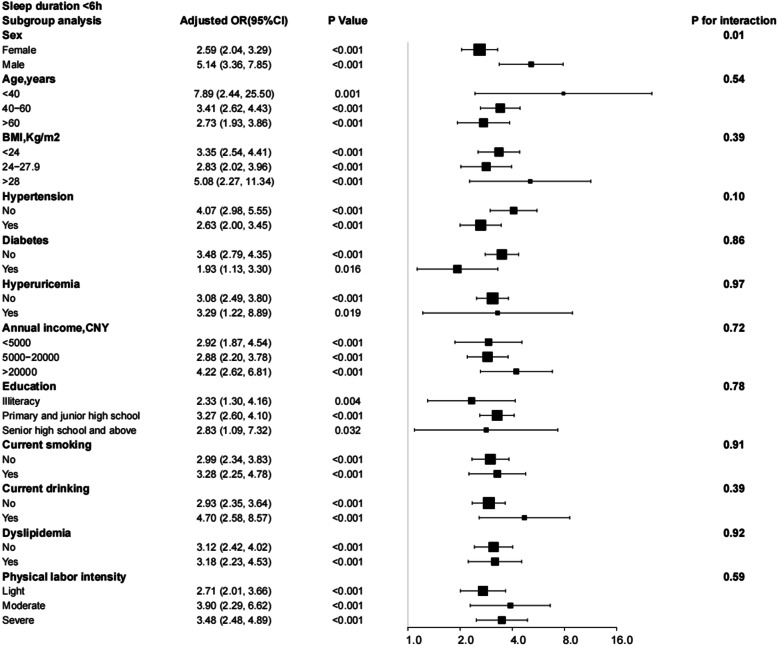
Subgroup analysis of the effect of sleep duration < 6 h on the risk of depression

**Fig. 5:**
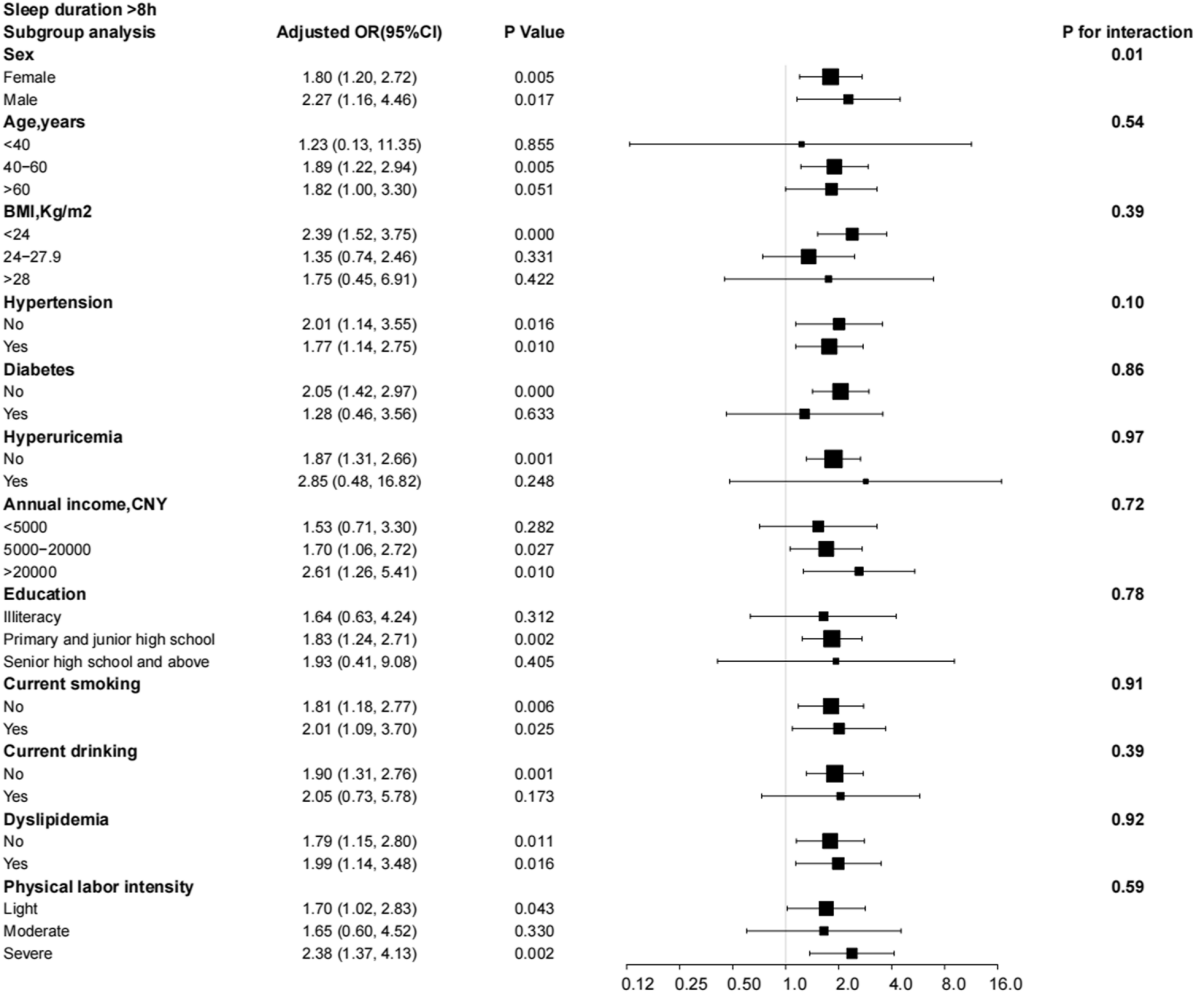
Subgroup analysis of the effect of sleep duration > 8 h on the risk of depression

**Fig. 6:**
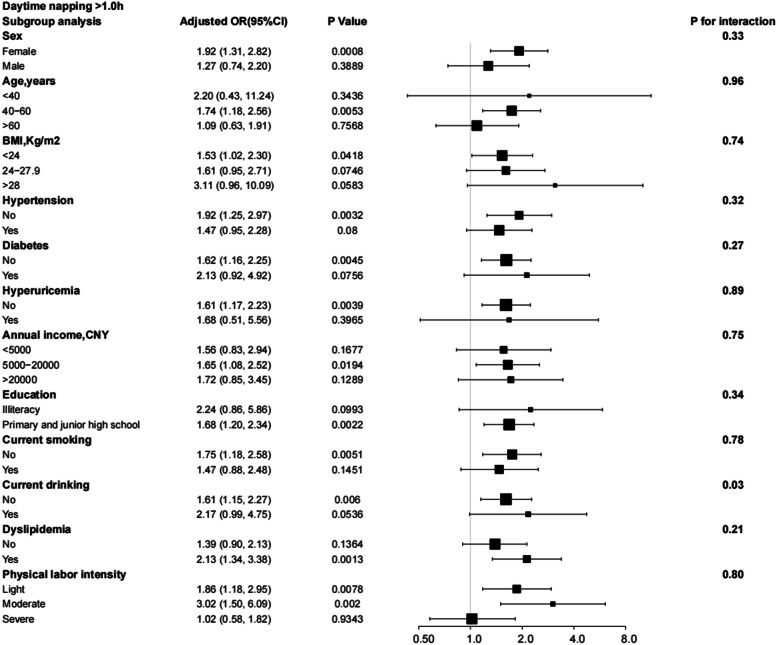
Subgroup analysis of the effect of daytime napping on the risk of depression

**Fig. 7:**
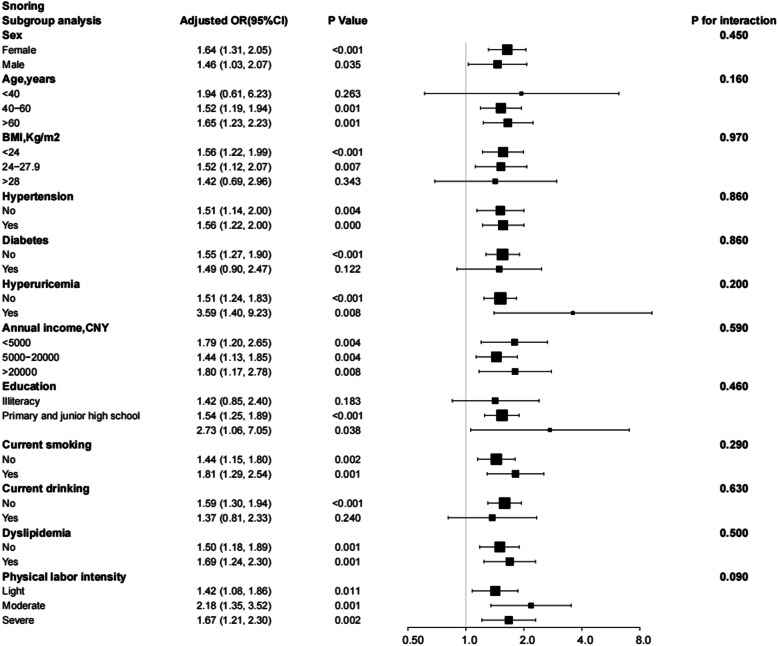
Subgroup analysis of the effect of snoring on the risk of depression

Subgroup analysis considered interactions of sleep duration, excessive daytime napping, snoring, and risk of depression with sex, age, BMI, hypertension, diabetes, hyperuricemia, annual income level, education, smoking, drinking, hyperlipidaemia, and physical labour intensity subgroups. After adjusting for all relevant risk factors, these interactions were not found to be significant. Except for sex (*P* for interaction = 0.01), the correlation between the risk of depression and < 6 h of sleep duration was consistent in all subgroups (Fig. 4). In comparison, > 8 h of sleep duration was only associated with an increased risk of depression in specific subgroups and interacts with sex (Fig. 5). Figure 6 shows that daytime naps > 1 h increased the risk of depression in specific subgroups (an interaction with drinking *P* for interaction = 0.03). In addition, in all stratified subgroups, the association between snoring and risk of depression was consistent (All *P* of interaction > 0.05; Fig. 7).

## Discussion

This cross-sectional study investigated the link between depression and sleep duration, daytime napping, and snoring. The results showed that short sleep duration (< 6 h), excessive sleep duration (> 8 h), excessive daytime napping (> 1 h), and snoring were significantly associated with an increased risk of depression, and sleep duration had a U-shaped relationship with depression risk.

Although previous studies found more evidence of the harmful effects of insufficient sleep on health, short sleep duration is also significantly related to an increased risk of depression [34]. Several previous studies of young people found that snoring [35] and insufficient sleep [36, 37] are related to depression. However, this study found similar results for people in rural China. In addition, the short duration of sleep may be attributed to chronic insomnia, which is highly comorbid with various psychological and physiological disorders and is considered a clear risk factor for depression [38]. However, regarding excessive sleep, some studies have indicated that prolonged sleep does not increase the risk of depression [14, 34]. Previous prospective studies of urban retirees in China also found no significant risk of new or recurrent depression associated with a long duration of sleep (> 9 h) [39], and patients with depression often had insufficient sleep [37]. In contrast, some studies have found that long sleep duration increases the incidence of depression [40–42]. We drew the same conclusion in the general rural population. Several possible reasons exist for these inconsistent results. First, different studies used different methods of adjusting for potential confounding factors. Second, the ethnicity and age of the participants were different. Third, because depression and sleep may have two-way effects [43], prospective cohort and cross-sectional studies may produce different findings. Fourth, differences in urban and rural areas, economic and social status, and occupation may significantly impact the pathogenesis of depression. This study was conducted in the relatively poor agricultural areas of Northeast China. The population of Liaoning Province in Northeast China is nearly 40 million, and 11.86 million people are involved in rural agriculture (according to the Liaoning Provincial Bureau of Statistics). In the end of 2013 (the year when the recruitment of this project ended), 2.14 million people in Liaoning Province had an annual per capita income of less than $441.2. In 2022, Liaoning Province’s per capita GDP was $9481.49, which was lower than the national per capita GDP of $12,814. However, a correlation exists between economic income and depression. Adverse financial events can easily lead to mental illness [3]. The uncertainty of agricultural cultivation in rural areas [44] and the relatively low medical expenditure coverage of rural cooperative medical insurance may play a role in mental illness. People living in poverty are usually more likely to be exposed to environmental irritants caused by pollution, extreme temperatures, and challenging sleep environments. Exposure to ambient air particles (such as more frequent sandstorms in Northeast China) increases the risk of mental disorders [45]. In addition, the frequent marginalization of the poor and economic depression in Northeast China may also lead to social isolation and loneliness in middle-aged and older people who stay in their hometown because most of their children go out to work [46], which is related to depression. Most participants in this study were farmers, with 54.5% of the population sleeping less than 6 h, significantly different from a previous finding, showing that 23.1% of urban Chinese participants reported short sleep duration [39]. These findings suggest that the population in this study may be relatively susceptible to depression. Therefore, unlike previous findings regarding the urban Chinese population, the findings of this study reveal that short sleep duration and excessive sleep may increase the risk of depression in the rural population.

Previous studies have revealed that sleep disorders [47] may cause changes in endocrine, metabolic, cardiovascular, and immune functions, which provide conditions for the occurrence and development of depression. Insufficient and excessive sleep are frequently related to chronic diseases such as obesity, diabetes, cardiovascular disease, and cancer [48]. Most of these chronic diseases are related to depression. An individual’s usual amount of sleep depends on their age and daytime alertness requirements. However, one-third of a person’s sleep need is determined by genetic inheritance [49]. Previous studies have also found that genes are inversely regulated by external factors such as actual sleep time and depression [50].

We also found that napping during the daytime was unrelated to depression; however, excessive napping time was an independent risk factor for depression. Although the underlying mechanism between daytime naps and depression remains unclear, longer naps may reflect a sedentary lifestyle [51]. Snoring increases the risk of depression in adolescents [52], but there is no definitive conclusion in adults. Nevertheless, it is generally believed that snoring is associated with diabetes [53], hyperuricemia [54], and insomnia [55], which are all risk factors for depression.

In addition, this study’s results support the importance of appropriate sleep duration or treatment for snoring in preventing depressive emotions. However, this study also has a few limitations. First, the results were based on cross-sectional data and could not suggest a causal relationship between the risk of depression and sleep patterns or snoring. Second, the information about sleep and snoring was based on self-reported measures, which might be susceptible to reporting biases. Third, we did not collect detailed information regarding the frequency or severity of snoring. Further studies are needed to analyse the link between depression and sleep duration and the severity of obstructive sleep apnoea and snoring. Additionally, a single assessment of a person’s sleep duration cannot account for variations in their sleep behaviour. For example, the duration of deep sleep and the number of awakenings during sleep may have an impact on the analysis results.

## Conclusions

Lack of nighttime sleep (short sleep duration), excessive sleep, and napping for more than one hour during the day were associated with a high risk of depression and had a combined effect with snoring. Successful implementation of sleep and snoring intervention measures may effectively encourage optimal sleep patterns and improve mental health in rural citizens.

## Data Availability

The datasets used/analysed during the current study are available from the corresponding author on reasonable request.

## Abbreviations

OR: Odds Ratio
CI: Confidence Interval
PHQ-9: Patient Health Questionnaire-9
SD: Standard Deviation
IQR: Interquartile Range
BMI: Body Mass Index
BP: Blood Pressure
SBP: Systolic Blood Pressure
DBP: Diastolic Blood Pressure
LDL-C: Low-Density Lipoprotein Cholesterol
HDL-C: High-Density Lipoprotein Cholesterol
Cr: Serum creatinine
UA: Uric Acid
eGFP: Estimated Glomerular Filtration Rate
GAM: Generalized Additive Model
